# Prevalence of Vitamin D insufficiency and low bone mineral density in elderly Thai nursing home residents

**DOI:** 10.1186/1471-2318-12-49

**Published:** 2012-09-02

**Authors:** Anuk Kruavit, La-or Chailurkit, Ammarin Thakkinstian, Chutintorn Sriphrapradang, Rajata Rajatanavin

**Affiliations:** 1 Medical Health Maintenance Organization, Eastern Health, Box Hill Hospital, Nelson Road, Box Hill, Melbourne, VIC 3128, Australia; 2 Department of Medicine, Faculty of Medicine, Ramathibodi Hospital, Mahidol University, Bangkok 10400, Thailand; 3 Research Center, Faculty of Medicine, Ramathibodi Hospital, Mahidol University, Bangkok 10400, Thailand; 4 Division of Endocrinology and Metabolism, Department of Medicine, Faculty of Medicine, Ramathibodi Hospital, Mahidol University, 270 Rama 6th Road, Bangkok 10400, Thailand

**Keywords:** Vitamin D, Osteoporosis, Bone density, Aged, Nursing homes, Collagen type I trimeric cross-linked peptide

## Abstract

**Background:**

Numerous emerging data from research on osteoporosis among Asians found differences from Caucasians. Therefore, the aim of this study was to determine the prevalence of vitamin D insufficiency and osteoporosis in elderly participants from two nursing homes in Thailand, a country located near the equator.

**Methods:**

The subjects of this cross-sectional study comprised 93 elderly Thai women who were living in institutional long-term nursing homes for the aged. Demographic data, daily food and calcium intake, physical activity, and sunlight exposure were measured. Lumbar spine and femoral neck bone mineral density (BMD) and biochemical levels including serum 25 hydroxyvitamin D [25(OH)D] and bone turnover markers were assessed. Vitamin D insufficiency was defined as 25(OH)D level < 70 nmol/l.

**Results:**

The mean age of subjects was 75.2 ± 6.0 (SD) years. Dietary calcium intake was low (322 ± 158 mg/day) The mean 25(OH)D level was 64.3 ± 14.9 nmol/L and the prevalence of vitamin D insufficiency was 38.7% (95% CI: 28.8%, 49.4%). There was no correlation between serum 25(OH)D concentrations and age (r = −.11, p = 0.3). The mean BMD of lumbar spine and femoral neck were 0.92 ± 0.19 and 0.65 ± 0.10 g/cm^2^, respectively. Nearly a half of the subjects had osteopenia (44.1%, 95% CI: 33.8%, 54.8%) and osteoporosis (47.3%, 95% CI: 36.9%, 57.9%). Circulating C-terminal telopeptide of type I collagen (CTx) level correlated significantly with both lumbar spine (r = −0.26, p = 0.01) and femoral neck BMD (r = −0.25, p = 0.02).

**Conclusions:**

More than one-third of Thai elderly women residing in nursing homes had vitamin D insufficiency. Almost all nursing home residents had osteoporosis and/or osteopenia.

## Background

Osteoporotic fracture has become a major global public health problem [1]. Many osteoporotic risk factors have been identified. Inadequacy of vitamin D is one of the important risks leading to fragility fracture. This condition results in abnormalities in calcium, phosphorus, and bone metabolism. It contributes to osteoporosis by secondary hyperparathyroidism and mineralization defect of the bone. In addition, vitamin D deficiency causes skeletal muscle weakness leading to more frequent falls, thereby increasing fracture risk [2,3].

The prevalence of vitamin D insufficiency or more seriously vitamin D deficiency has increased across the world. Even in countries with plentiful sunlight, vitamin D inadequacy was commonly found in 64% of postmenopausal women with osteoporosis [4]. Senior citizens are at risk for vitamin D deficiency because of poor dietary vitamin D intake and decreased exposure to sunlight. Aging also decreases the amount of 7-dehydrocholesterol produced in the skin by as much as 75% by the age of 70 years [5,6]. Prevalence studies of osteoporosis and vitamin D status in nursing home residents have been widely investigated in Western countries [7-9]. Numerous emerging data from research on osteoporosis among Asians found differences from Caucasians [10]. Data in Asian countries, especially South East Asian region, are still lacking.

We conducted a cross-sectional study which aims to measure vitamin D level and bone mineral density (BMD) in elderly Thai subjects who live in nursing homes.

## Methods

### Subjects

A cross-sectional study was conducted of 93 women aged 61–97 years in elderly care institutions within the vicinity of Bangkok (38 from Ban-Bangkae 1 and 55 from Ban-Bangkae 2) which are located in the West of Bangkok at the latitude of 13°45′N. These two nursing homes are Thai institutions established to provide elderly care services. Residents can either choose to live in a dormitory free of charge or in a single room with monthly payments. All meals are provided by the nursing home. However, the elderly can choose the amount of food intake they want. Before living in a nursing home, applicants have to make a reservation well in advance. Physicians from the government hospitals visit the elderly on the regular basis. These medical services are free of charge.

Thailand is situated in a geographic area where the sun shines almost all year round during the day causing little of the seasonal fluctuations seen in countries where there is a winter season. The average duration of sunshine in Bangkok is 4.7 to 8.3 hour per day. The minimum and maximum temperature in Bangkok ranges from 17.9°C to 38.1°C (Thai Meteorological Department, 2008). The study was conducted between November and February, during the cool season in Thailand. Participants were excluded if they received estrogen therapy, any medications influencing bone metabolism (such as bisphosphonate) within the previous six months, glucocorticoid, anticonvulsant or fluoride within one year, had overt hyperparathyroidism, were unwilling to participate with the study or unable to give consent. All the subjects were transferred by a van, and were accompanied by the investigators at all times from Ban-Bangkae to Ramathibodi Hospital, University Hospital in Bangkok for investigations.

Demographic data such as age, height and weight were collected. Body mass index (BMI) was calculated as the body weight in kilograms divided by the square of height in meters. Information on menopausal age, present illness, medication, vitamin and calcium supplements and other lifestyle information including alcohol consumption and smoking habits were obtained through an interview. Three (3.2%) and 4 (4.3%) of the women had a history of previous hip and wrist fractures, respectively. No subjects had a previous history of vertebral fracture. The study was approved by the Ethics Committee of Ramathibodi hospital. Written informed consent was obtained from all of the participants.

### Dietary calcium intake, physical activity, and sunlight exposure

A 3-day food record was used to estimate the daily food intake including calcium intake. All food records were analyzed for nutrient intake by using INMUCAL software which can calculate nutritional value from various Thai foods [11]. Energy expenditure per day was calculated on the basis of energy requirement for each activity expressed in terms of metabolic equivalents (METs, ratio of working and resting working metabolic rate) [12]. Daily sunlight exposure was quantified based on the interview questions on frequency and length of outdoor activities, sunscreen use, and usual outdoor attire.

### Biochemical measurement

Fasting blood samples were drawn in the morning between 8.00-10.00 am and kept in the -80°C freezer for biochemical analysis later. Twenty-four hour urine for calcium was obtained. Urinary creatinine was also measured to check completeness of collection. Serum calcium, creatinine, inorganic phosphorus, total alkaline phosphatase and urinary calcium were analyzed on an automated biochemical analyzer (Dimension RxL, Dade Behring Co Ltd, USA). Serum 25-hydroxyvitamin D [25(OH)D] was measured by radioimmunoassay (DiaSorin Inc., Stillwater, MN, USA) with an intra-assay precision of 8.9%. Plasma intact parathyroid hormone (PTH) and serum C-terminal telopeptide of type I collagen (CTx) levels were determined by electrochemiluminescence immunoassay on an Elecsys 2010 analyzer (Roche Diagnostic GmbH, Mannheim, Germany). The assays have intra-assay precision of 3.6% and 5.4%, respectively.

Vitamin D insufficiency was defined as a serum 25(OH)D concentration ≤ 70 nmol/l which was the threshold level at which serum PTH began to rise in elderly Thai women [13].

### Bone mineral density (BMD)

BMD of lumbar spine at L2-4 and femoral neck was measured by dual-energy X-ray absorptiometry (DXA) (Lunar Expert XL, Lunar Corp, USA). Subjects were classified as having osteoporosis if the BMD T-score was ≤ -2.5, or as having osteopenia if the BMD T-score was > −2.5 and < −1.0, or as normal BMD if the BMD T-score was ≥ -1.0, according to the World Health Organization (WHO) criteria [14].

### Statistical analysis

Descriptive results were presented as mean ± SD or median and 95% confidence interval of median. The chi-square test was used to compare proportions between groups. A pairwise correlation was assessed using Pearson correlation coefficient. Stepwise multiple linear regression was applied by fitting BMD on significant variables (i.e. years since menopause, energy expenditure, serum calcium, serum CTx and urinary calcium/urinary creatinine) suggested by the univariate analysis.

Association between CTx and osteoporosis was explored using a receiver operating characteristic (ROC) curve analysis and the cut-off value for serum CTx in classifying osteoporosis was then determined. A p-value less than 0.05 was considered as statistically significant. All statistical analyses were performed using the SPSS package version 15.0 (SPSS Inc., Chicago, Illinois, USA).

## Results

**Figure 1:**
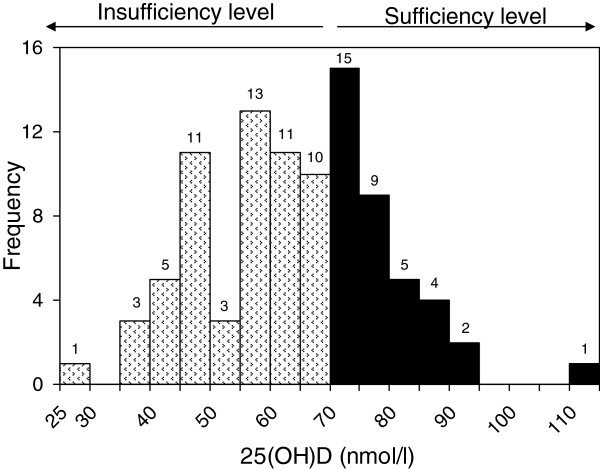
Frequency distribution of serum 25(OH)D concentrations of the subjects.

Ninety-three women aged 61 to 97 years with normal renal (creatinine < 1.5 mg/dl) and hepatic functions (AST/ALT level < 2-fold of upper normal limit) were included in the study. Demographic and biochemical variables of subjects are summarized in Table 1. All participants in this study were non-smoking and non-alcohol drinking women with the mean age of 75.2 ± 6.0. Among 93 participants, only 16% of subjects were directly exposed to more than 2 hours of sunlight per day. Twenty-seven (29%) and 9 (9.7%) of subjects received elemental calcium 240 mg/day and/or alfacalcidol 0.25 mg/day for less than 3 months. Only six women (6.5%) received both calcium and vitamin D supplementation. The purpose of these supplements was to prevent osteoporosis. However, serum 25(OH)D level was not different between women who were taking vitamin D supplement and those who were not (66.2 ± 16.0 vs. 64.1 ± 14.9 nmol/l). The frequency distribution of 25(OH)D concentration of the subjects is shown in Figure 1. The distribution was approximately normal with a minimum value of 26.8 nmol/l. The prevalence of vitamin D insufficiency was 61.3% (95% CI: 50.6%, 71.2%). Only thirty-six women (38.7%) had 25(OH)D levels > 70 nmol/l, which has recently been proposed as the optimal concentration in elderly Thai citizens [13]. However, when using the threshold as defined by 25(OH)D levels less than 50 [15] and 75 nmol/L [16], the prevalence of vitamin D insufficiency was 21.5% and 77.4%, respectively.

**Table 1:** Demographic and Biochemical Variables of the Subjects (N = 93)

**Characteristics**	**Mean ± SD**	**Reference range**
Age (years)	75.2 ± 6.0	
Weight (kilograms)	52.5 ± 8.9	
Height (centimeters)	147.7 ± 6.1	
BMI (kg/m^2^)	24.1 ± 3.6	
Years since menopause (years)	28.9 ± 8.0	
Energy expenditure (kcal/day)	1,589.4 ± 285.4	
Dietary calcium intake (mg/day)	322.3 ± 158.4	
Caloric intake (kcal/day)	861.4 ± 296.2	
Serum calcium (mmol/L)	2.4 ± 0.08	2.2 -2.62
Serum creatinine (μmol/l)	70 (44–151)^*^	53 - 88
Inorganic phosphorus (mmol/L)	1.17 ± 0.13	0.81 – 1.58
Alkaline phosphatase (U/l)	82.7 ± 25.7	50 - 136
Serum 25(OH)D (nmol/L)	69.3 ± 15.4	> 70
Plasma intact PTH (pmol/L)	4.3 ± 1.5	1.6 – 6.9
Serum CTx (ng/ml)	0.42 ± 0.21	0.104 – 1.008
Urinary calcium /urinary creatinine (mmol)	0.48 ± 0.29	

* = median (range), BMI = body mass index, 25(OH)D = 25-hydroxyvitamin D, PTH = parathyroid hormone, CTx = C-terminal telopeptide of type I collagen.

The mean BMD of lumbar spine and/or femoral neck were categorized in the osteopenia or osteoporosis with a prevalence of 41 (44.1%, 95% CI: 33.8, 54.8%) and 44 (47.3%, 95% CI: 36.9%, 57.9%), respectively (Table 2). The prevalence of osteoporosis or osteopenia at the femoral neck was significantly higher than the prevalence at the lumbar spine (89.2% vs. 73.1%, p = 0.019).

**Table 2:** Classification of BMD Status (N = 93)

**BMD**	**Status**	**Number (%)**	**g/cm**^**2**^**,**	**T-score,**
			**mean ± SD**	**mean ± SD**
Lumbar spine at L_2- 4_	Normal	25 (26.9)	1.16 ± 0.16	0.32 ± 1.29
	Osteopenia	42 (45.2)	0.89 ± 0.05	−1.88 ± 0.44
	Osteoporosis	26 (28.0)	0.72 ± 0.09	−3.36 ± 0.76
Femoral neck	Normal	10 (10.8)	0.84 ± 0.07	−0.46 ± 0.58
	Osteopenia	51 (54.8)	0.67 ± 0.05	−1.90 ± 0.45
	Osteoporosis	32 (34.4)	0.55 ± 0.04	−2.94 ± 0.37

BMD = bone mineral density.

Pearson correlation was applied to explore the correlation between variables and markers. Serum 25(OH)D level did not correlate with age (r = −.11, p = 0.3) and none of the other variables. Energy expenditure and serum CTx correlated significantly with both lumbar spine (energy expenditure, r = 0.24, p = 0.02; CTx, r = −0.26, p = 0.01) and femoral neck BMD (energy expenditure, r = 0.22, p = 0.03; CTx, r = −0.25, p = 0.02), whereas urinary calcium and urinary creatinine ratio correlated significantly with lumbar BMD (r = −0.27, p = 0.008). In addition, serum CTx correlated with serum PTH levels. Factors that correlated significantly with BMD in the univariate analysis were simultaneously entered in a linear regression model to identify the determinants of BMD by stepwise selection procedure. We found that serum CTx concentration was the major determinant of both lumbar spine and femoral neck BMD, i.e., every 1 unit of CTx increased, lumbar and femoral BMD decreased 0.226 and 0.121 g/cm^2^ (Table 3). The ROC curve analysis was applied to determine a CTx cut-off in discriminating osteoporosis. A cut-off value of 0.237 ng/ml was chosen, which had a sensitivity of 81% and a specificity of 22%.

**Table 3:** Independent determinant of BMD by sites: multiple linear regression

**Variables**	**Lumbar spine at L2-L4 BMD (g/cm**^**2**^**)**			**Femoral neck BMD (g/cm**^**2**^**)**		
	**Coefficients**	**SE**	**P-value**	**Coefficients**	**SE**	**P-value**
Intercept	1.093	0.052	<0.001	0.773	0.044	<0.001
Serum CTx (ng/ml)	−0.226	0.091	0.015	−0.121	0.050	0.017
Urinary calcium/ urinary creatinine (mmol)	−0.172	0.066	0.011	exclude	-	-
Year since menopause (years)	exclude	-	-	−0.003	0.001	0.048
Energy expenditure (kcal/day)	exclude	-	-	exclude	-	-

BMD = bone mineral density, CTx = C-terminal telopeptide of type I collagen.

## Discussion

Senior citizens in nursing homes often have limited access to direct sunlight because of mobility issues. Nevertheless, Thailand is located near the equator where sufficient sunlight is available to provide adequate ultraviolet B exposure all year round. Elderly Thai nursing home residents were expected to have less prevalence of vitamin D insufficiency compared with people in countries located at high latitudes. However, mean serum level of 25(OH)D in the elderly nursing home residents in this study was 65 nmol/L and 61.3% of them had vitamin D insufficiency (≤ 70 nmol/l). The level of 25(OH)D in the present study was lower than the level in a population of younger normal health-conscious Thai women (73–129 nmol/L) [17]. In addition, there were no difference of the mean serum 25(OH)D concentration between those women taking and those not taking vitamin D supplement. This may result from the limited number of vitamin D supplement subjects in the group. The mean serum 25(OH)D level of elderly Thai nursing home residents was still higher than populations in countries where fortification of milk and vitamin D supplements is common such as in the United States (mean 25(OH)D level = 30 nmol/l) [18]. Elderly people in Japan where consumption of oily fish high in vitamin D is part of the usual diet, had the mean 25(OH)D as low as 29.9 nmol/l [19]. This evidence supports the role of sunlight exposure in elderly people to improve vitamin D status.

Interestingly, dietary calcium intake did not correlate with either lumbar spine or femoral BMD in this population. This may be explained by the fact that all residents are provided with the same food each meal. Mean calcium intake was 322 ± 158 mg/day, which was higher than previously report in Thai farmers (236 ± 188 mg/day) [20] and was similar to other populations of Thai urban postmenopausal women (348.9 ±12.9 mg/day) [21]. It is much lower than that reported in a Caucasian population (670 ± 258 mg/day) [22]. Lactose intolerance can be a barrier to milk consumption among Asians leading to the consumption of fewer calcium-containing foods than Caucasian women [23]. The high normal level of serum PTH in this study (mean 4.3, normal range 1.1 - 5.8 pmol/L) can be partially explained by the low calcium intake and less calcium intestinal absorption in the elderly. Moreover, a significant positive relationship between serum PTH and CTx levels was found. The US National Institute of Health recommends the daily calcium requirement to be as high as 1,500 mg calcium in postmenopausal women not using hormone replacement therapy and in both men and women aged over 65 years [24]. However, the addition of 500 mg/day of elemental calcium would be optimal to achieve maximum calcium retention in Asians as demonstrated by a balanced study in Japanese postmenopausal women [25]. Seniors living in nursing homes should be encouraged to increase outdoor activities and sunlight exposure with additional consumption of calcium-rich food or calcium and vitamin D supplement to reduce the risk of osteoporotic fracture.

Serum 25(OH)D levels had an inverse relationship with age in previous reports [26,27]. However, age-related decline in serum 25(OH)D was not consistent in other reports or in the present study [19,28,29]. In addition, an inverse association between serum 25(OH)D and PTH levels was not observed in our study. This might be explained by the limited number of subjects with low vitamin D insufficiency (N = 36; 38.7%). Furthermore, there was no association between vitamin D or PTH levels and BMD. This result is similar to previous reports on Asian women [30,31]. On the other hand, European and North American studies have shown a correlation between serum 25(OH)D concentration and vertebral or femoral BMD [32,33]. From a previous study, positive relationship between serum 25(OH)D and femoral neck BMD will only be significant when serum 25(OH)D is lower than 30 nmol/l [34]. In our population there was only one subject with 25(OH)D level less than 30 nmol/L.

Based on the WHO classification, the prevalence of osteoporosis among nursing home residents was 47.3%. Percentage of osteoporosis at femoral neck was higher than at the lumbar spine. This prevalence was relatively lower than other studies in a Western population that varied from 55% to 85.8% [35,36]. Besides genetic differences and life style factors, a selection bias might lead to lower prevalence of osteoporosis because the eligibility criteria in this study excluded all subjects who were not healthy.

Factors associated with low BMD were identified in this study. Both energy expenditure and serum CTx were correlated with lumbar and femoral neck BMD. The positive effect of high physical activity to increased BMD and reduced osteoporotic fracture rate has been shown in various studies for many different ethnic groups [37]. CTx is a bone resorption marker, and has been shown to be a convenient tool for monitoring BMD after treatment [38]. In a previous report, serum CTx level was a better indicator than other bone markers, providing an earlier indication of response [39]. BMD measurement is relatively expensive and may not be accessible to all individuals. Serum CTx level could use as an alternative screening and monitoring method for individuals who may be suffering from osteoporosis. This strategy was proposed because it was more convenient for nursing home residents who cannot easily travel to the hospitals where BMD measurement facilities are available. A CTx cut-off value for this nursing home population was found to be 0.237 ng/ml with a sensitivity of 81%. This implication applies to elderly persons who have a serum CTx level higher than 0.237 ng/ml; they should be monitored more closely for the presence of osteoporosis such as arranging for the more expensive BMD scan. However, the cost-effectiveness should be evaluated in the future.

### Limitations

A number of limitations exist with respect to the present study. Firstly, this is a cross-sectional study; the correlations cannot imply the causation relationships between parameters and low BMD. Moreover, the study could not include all the residents who resided in Thai nursing homes. The data represent only subjects who met the eligibility criteria and agreed to participate. Urine samples may not be adequately collected due to the inconvenience of the procedure. However, this limitation had been corrected by expressing data as a ratio relative to urine creatinine. Furthermore, energy expenditure of the elderly subjects was obviously higher than the energy intake. Energy expenditure per day was calculated on the basis of energy requirement for each activity expressed in terms of METs. The high energy expenditure result might be overestimated due to the formula of the MET, which is more suitable for young adults whereas the subjects in this study are elderly. This could be explained from the previous study that overestimation of energy expenditure was greater with older age [40]. Lastly, there were 9 subjects who had been taking active form of vitamin D supplements for about 3 months. They were not excluded because their 25(OH)D levels were not different from the rest.

## Conclusions

About one-third of elderly women residing in nursing homes had vitamin D insufficiency. Almost all the nursing home residents had osteoporosis and/or osteopenia.

## Competing interests

The authors declare that they have no competing interests.

## Authors’ contributions

Study concept and design: R, T. Acquisition of subjects and data: K, C. Analysis and interpretation of data: K, T, C, R, S. Preparation of manuscript: K, C, R, S. All authors read and approved the final manuscript.

## Pre-publication history

The pre-publication history for this paper can be accessed here:

http://www.biomedcentral.com/1471-2318/12/49/prepub
